# Automatic Segmentation of Human Cortical Layer-Complexes and Architectural Areas Using *Ex vivo* Diffusion MRI and Its Validation

**DOI:** 10.3389/fnins.2016.00487

**Published:** 2016-11-10

**Authors:** Matteo Bastiani, Ana-Maria Oros-Peusquens, Arne Seehaus, Daniel Brenner, Klaus Möllenhoff, Avdo Celik, Jörg Felder, Hansjürgen Bratzke, Nadim J. Shah, Ralf Galuske, Rainer Goebel, Alard Roebroeck

**Affiliations:** ^1^Department of Cognitive Neuroscience, Faculty of Psychology and Neuroscience, Maastricht UniversityMaastricht, Netherlands; ^2^Research Centre Jülich, Institute of Neuroscience and Medicine (INM-4)Jülich, Germany; ^3^Department of Biology, TU DarmstadtDarmstadt, Germany; ^4^Department of Forensic Medicine, Faculty of Medicine, Goethe University FrankfurtFrankfurt, Germany; ^5^Department of Neurology, Faculty of Medicine, Jülich Aachen Research Alliance, RWTH Aachen UniversityAachen, Germany; ^6^Department of Neuroimaging and Neuromodeling, Netherlands Institute for Neuroscience - KNAWAmsterdam, Netherlands

**Keywords:** diffusion MRI, cortical layers and areas, ultra-high field MRI, MR-based histology, histological validation

## Abstract

Recently, several magnetic resonance imaging contrast mechanisms have been shown to distinguish cortical substructure corresponding to selected cortical layers. Here, we investigate cortical layer and area differentiation by automatized unsupervised clustering of high-resolution diffusion MRI data. Several groups of adjacent layers could be distinguished in human primary motor and premotor cortex. We then used the signature of diffusion MRI signals along cortical depth as a criterion to detect area boundaries and find borders at which the signature changes abruptly. We validate our clustering results by histological analysis of the same tissue. These results confirm earlier studies which show that diffusion MRI can probe layer-specific intracortical fiber organization and, moreover, suggests that it contains enough information to automatically classify architecturally distinct cortical areas. We discuss the strengths and weaknesses of the automatic clustering approach and its appeal for MR-based cortical histology.

## Introduction

Although there has been a century-old dominance of the cytoarchitectonic classification of human cortex, mostly based on Brodmann's cortical parcellation scheme (Brodmann, 1909), there is currently a renewed appreciation for the richness of intra-cortical detail visible in its myeoloarchitecture (Nieuwenhuys, 2013). This renewed interest is driven in part by clear indications that several magnetic resonance imaging (MRI) contrasts (e.g., T1 and T2^*^) in gray matter show sensitivity to myelin content (Fatterpekar et al., 2002; Duyn et al., 2007; Geyer et al., 2011; Barazany and Assaf, 2012; Lee et al., 2012; De Martino et al., 2015), which has ignited ambitions of MRI-based histology, possibly even *in vivo* (Dick et al., 2012; Deistung et al., 2013; Sereno et al., 2013; Truong et al., 2014; Turner and Geyer, 2014). Diffusion magnetic resonance imaging (dMRI), a technique mostly used to date to probe brain white matter, has recently also started to be applied to gray matter, both *ex vivo* (D'Arceuil and de Crespigny, 2007; D'Arceuil et al., 2007; Miller et al., 2011; Kleinnijenhuis et al., 2013; Dell'Acqua et al., 2013; Leuze et al., 2014; Aggarwal et al., 2015) and *in vivo* (Heidemann et al., 2012; McNab et al., 2013; Nagy et al., 2013; Kleinnijenhuis et al., 2015). When applied to post mortem samples, in particular, high spatial resolution dMRI has been used to highlight different layers or layer-complexes in human cortical gray matter (Oros-Peusquens et al., 2012; Roebroeck et al., 2012; Bastiani et al., 2013; Kleinnijenhuis et al., 2013; Leuze et al., 2014; Aggarwal et al., 2015). This results from the sensitivity of dMRI to characteristic radial and tangential orientations of myelinated and unmyelinated axons and dendrites (i.e., neurites). These studies have shown that at high enough spatial resolution cortical layers can be manually delineated based on the average organization of local neurite orientation and this lamination can be distinguished between preselected cortical areas. This suggests dMRI's specific sensitivity to radial and tangential neurite orientations may enable architectonic characterization of cortex akin to myeloarchitecture. Beyond manual delineation of cortical layer and areas boundaries, dMRI's well-structured 3D data seems to lend itself to automatic clustering of architectural properties and localization of boundaries (Nagy et al., 2013).

In this work we investigate whether high-resolution dMRI data acquired *post mortem* can support automatic segmentation of human cortical layer-complexes and area boundaries in human cortex by unsupervised clustering of their diffusion characteristics. First, we investigate whether groups of consecutive layers (or layer-complexes) can be distinguished in human primary motor and premotor cortex. Second, we investigate whether the signature of diffusion MRI signals over cortical depth can be used as a criterion to detect area boundaries. We validate these automatic dMRI based classifications *in situ* by histological analysis of the same tissue and test the reproducibility of the results over repeated acquisitions at different temperatures. We conclude with a discussion of the strengths and weaknesses of the automatic clustering approach, including requirements on resolution and field-of-view of diffusion acquisitions and the variation of layer position and thickness with cortical curvature.

## Materials and methods

### Tissue preparation

This study, consisting of two full dMRI acquisitions and histology on several sections, was performed on a block of human brain tissue (38.94 × 36.3 × 23.76 mm) which comprised parts of primary motor and medial and lateral premotor cortex. The tissue was obtained 6 h post mortem from the left hemisphere of a female subject, aged 38, without known neurological or psychiatric disorders. All procedures were approved by the ethical committee of the University Clinic, Frankfurt/M, Germany. Under this approval, the use of the tissue did not require the consent of the relatives, as the sample was obtained for routine forensic studies and could not be placed back in the body at the end of the autopsy. The tissue was prepared and fixed for 48 h using a solution containing 2.6% paraformaldehyde, 0.8% iodoacetic acid, 0.8% sodium periodate, and 0.1 M D—L -lysine in 0.1 M phosphate buffer at pH 7.4 at 4°C. Thereafter, the tissue was stored in a solution containing 2% paraformaldehyde in 0.1 M phosphate buffer at pH 7.4 at 4°C. MR scans were performed after about 1 year of fixation. The tissue was scanned immersed in the fixation solution to ensure long-term preservation for subsequent histological processing.

### MR data acquisition

Two full dMRI acquisitions, each about 120 h long, were performed. Measurements on a small-bore 9.4T system equipped with a 12 cm ID, 600 mT/m, 100 μs rise time gradient coil and interfaced to a Siemens Tim Trio console. A 7 cm loop coil was used for RF transmission and signal reception (Supplementary Figure 1). A 2D spin-echo sequence was modified to include a diffusion preparation module and implement pulsed gradient spin echo (PGSE or Stejskal-Tanner) diffusion MR imaging. The measurement parameters were: FOV 53 × 60 mm^2^, matrix 156 × 176, 97 contiguous slices (achieving isotropic resolution of 340 μm^3^), TR = 10,000 ms, TE = 45 ms, Δ = 22.5 ms, δ = 3 ms, |G| = 466 mT/m, flip angle = 90°, 4 averages, b = 3000 mm^−2^s, 60 diffusion encoding directions (obtained by an electrostatic repulsion algorithm on the whole sphere) and six b = 0 acquisitions. High *b*-values will increase diffusion contrast in a dMRI study. However, in post-mortem dMRI studies, despite having high-amplitude gradient sets available, the achievable *b*-value is mainly limited by the shorter T2 of both white and gray matter due to fixation. As a consequence, echo times have to be much shorter than those used for *in vivo* studies to obtain a reasonable SNR, especially at high resolution. In the present study, therefore, we chose to set the *b*-value to 3000 mm^−2^/s. This allowed us to obtain mean SNRs (calculated as the mean divided by the standard deviation of the b0 signal in each voxel) of 27.2 and 32.7 through the whole tissue sample for the two datasets, respectively. Other studies have used similar *b*-value ranges, slightly lower when trying to achieve a higher resolution (Leuze et al., 2014) or slightly higher when stronger magnets and gradient sets are available (Kleinnijenhuis et al., 2013). Acquisitions for dataset 1 were performed at room temperature (average 24°C inside the bore). Data acquisition was repeated after 1 week to test reproducibility under different diffusion conditions. Therefore, during the second scanning session (dataset 2), the temperature in the scanner was raised to 30°C using an in-bore hot air animal warming system and constantly monitored with a temperature probe. All other acquisition parameters for dataset 2 were the same.

### Diffusion MRI data analysis

Diffusion weighted datasets 1 and 2 were preprocessed in order to correct for image shift and geometric distortions arising from eddy currents induced by diffusion gradients using the FMRIB's Diffusion Toolbox available in FSL (Jenkinson et al., 2012). The estimated transformation matrices were used to rotate the diffusion gradient directions accordingly (Leemans and Jones, 2009) and perform corrections for the corresponding signal magnitude by a normalization using the determinant of the Jacobian matrix. Manual segmentation of the averaged non-diffusion-weighted (i.e., pure T2-weighted or b0) volumes was performed to obtain white and gray matter masks. Affine registration (12 degrees of freedom) from dataset 1 to dataset 2 was performed using the FLIRT toolbox (Jenkinson and Smith, 2001; Jenkinson et al., 2002) available in FSL. That was done to make sure, mainly, that differences in susceptibility distortions and, secondly, minor changes in the tissue sample size and shape (due to e.g., expansion with temperature change) were corrected. The SNR of the two datasets was calculated as the mean divided by the standard deviation of the b0 signal in each voxel. Mean SNRs were 27.2 and 32.7, for dataset 1 and dataset 2, respectively, with a standard deviation of 12 and 13.2. For white matter, the mean SNR was 19.4 (std = 6.7) and 24.2 (std = 8.1) in the two datasets respectively. For gray matter, the mean SNR was of 33.5 (std = 11.7) and 41.6 (std = 11.6) in the two datasets, respectively.

### Histology

After MRI scanning, the block was cut in half, with the cutting plane approximately parallel to the xy plane of the scan. The anterior part was sectioned at a slice thickness of 60 μm using a microtome (Supercut 2050, Reichert–Jung) equipped with a freezing stage (Frigomobil, Leica Microsystems, Wetzlar, Germany). In order to improve orientation within the sliced tissue material later on, blockface photos were taken every second slice, as in Choe et al. (2011). This procedure resulted in 343 sections, from which every 5th was stained for myelin using the Gallyas method (Gallyas, 1979), giving 69 myelin stained sections for analysis. Furthermore, every 20th slice was stained for cell bodies with cresyl violet to allow for laminar and areal classification by cytoarchitecture. The sections stained for myelin were digitized using a high-resolution microscope setup (AxioImager Z1, Carl Zeiss AG, Oberkochen, Germany) equipped with a motorized stage and high resolution camera (Axiocam HRm, Carl Zeiss AG, Oberkochen, Germany). Images were obtained at 50x magnification and assembled using the MosaiX recording technique (Carl Zeiss AG, Oberkochen, Germany). The resulting images were monochrome at 8 bit depth and a pixel resolution of 1.3 μm. Based on the anatomical descriptions provided by Von Economo and Koskinas (1925) and Sanides (1962), the analyzed tissue was identified as primary motor and lateral premotor cortex on the basis of its cytoarchitecture. Likewise, the analysis of the myelin stained sections confirmed our cytoarchitectonic classification using the criteria provided by Nieuwenhuys (2013) and Vogt (1910). Subsequently cortical layers were delineated on both a cytoarchitectural and myeloarchitectural basis. To align digitized 2D histological sections with the 3D dMRI data, the alignment procedure described in Seehaus et al. (2013) was used.

### Cortical layer demarcation

The cortical surface and cortical depth were sampled by surface reconstruction of the white/gray matter and pial boundary and discretizing cortical depth in steps between these. Gray matter volume was sampled by nine different meshes of 60,000 vertices each at fixed local cortical depth steps (10–90%, 10% step size) between the white/gray matter boundary (0%) and the pial boundary (100%). The outer white/gray matter and the pial boundaries themselves were avoided to avoid partial volume effects with white matter tissue and embedding fluid. The depth sampling technique is based on Laplace's equation (Jones et al., 2000; Zimmermann et al., 2011) as implemented in BrainVoyager QX (Goebel et al., 2006; De Martino et al., 2013). This technique initially sets two different voltage values at the aforementioned boundaries and computes the smoothed transitional voltages between them. As a result of this initialization step, voltage gradients can be calculated in every voxel of the entire gray matter volume. Integrating across this gradient vector field results in the definition of streamlines which can be used for cortical depth sampling.

Features used to classify cortical layers were derived from the apparent diffusion coefficient (ADC) profile. Samples over a whole sphere of the ADC were obtained from the high angular resolution imaging (HARDI) acquisitions, computed as:

$$\begin{array}{l} ADC_{i}\;=\;-\frac{1}{b}\log\left(\frac{S_{i}}{S_{0}}\right) \end{array}$$

where *ADC*_*i*_ is the coefficient calculated for the ith diffusion gradient direction, *b* is the b-value in s^*^mm^−2^, *S*_*i*_ is the measured signal when applying the ith diffusion encoding gradient and *S*_0_ is the b0 signal without diffusion weighting. A 6th order spherical harmonic basis (capable of representing multiple fiber populations) was fitted to the ADC profile at each point of the 9 GM surfaces, applying trilinear interpolation in each diffusion-weighted image. The spherical harmonic basis comprised only even terms as diffusion is modeled as having an equal contribution along opposite directions (therefore we prefer to talk about orientations). Tests of fit (by sum-of-squared error, SSE) and model comparison (Akaike information criterion, AIC) were performed for spherical harmonic orders of 2, 4, 6, and 8 (Supplementary Figure 2). The choice for order 6 was motivated by a trade-off between number of parameters estimated, data-fit, and model comparison parameters. A total of 28 unique coefficients needs to be estimated when using spherical harmonics up to the 6th order as a basis function set to represent the acquired signal, which gives about 2 data points per estimated parameter. Data fit progressively increased from order 2 to order 8, as expected from the increasing number of coefficients. Based on SSE alone, order 8 might be preferred. However, this would involve estimating a number of parameters close to the number of datapoints (45 for order 8). Looking at the AIC for a series of maximum harmonics orders show that there is no difference when modeling the acquired diffusion signal using either 6 or 8. Therefore, a maximum order to 6 was set as a tradeoff between the achieved goodness of fit and number of parameters. This both limited the total number of parameters to be estimated (28 for order 6 rather than 45 for order 8) while keeping the SSE distribution smaller than when using low orders fit (e.g., 2 or 4). Moreover, low SH orders would not be capable of representing both radial and (multiple) tangential diffusion fiber orientation distribution (FOD) peaks.

Local surface normals, estimated at every reconstructed surface point, where then used to reconstruct radial cortical depth profiles and each ADC profile was then interpreted in the local “cortical coordinate system” (cf. McNab et al., 2013) by rotating the cortical radial orientation to the z-axis. In this coordinate system four (groups of) features were derived (Nagy et al., 2013): (i) the average of the entire spherical ADC profile (total cortical diffusivity, 1 feature), (ii) the value of the ADC profile along the local surface normal (radial cortical diffusivity, 1 feature), (iii) the average of the ADC profile over the cortical tangential plane (tangential cortical diffusivity, 1 feature), and (iv) the even spherical harmonics coefficients of the ADC profile (28 features). A feature space containing these indices was constructed, resulting into a 60,000 (vertices) × 9 (surfaces) × 31 (features). For the voxel-by-voxel layer clustering this space was transformed into a (60,000^*^9) × 31 feature matrix and then fed into an unsupervised *k*-means clustering algorithm to classify cortical locations into different cortical layers. The clustering algorithm was implemented using the statistics toolbox in Matlab (R2010b, The MathWorks, Natick, Massachusetts, USA) and tries to cluster each point in the multi-feature space around *k* centroids by minimizing squared Euclidian distances between each point and the proposed centroids.

The number of clusters (i.e., centroids) *k* to be identified was set on the basis of a silhouette index analysis, taken from the statistics toolbox in Matlab (R2010b, The MathWorks, Natick, Massachusetts, USA). The silhouette index is a measure of how well a certain element is represented by a certain cluster (Rousseeuw, 1987). It is calculated using the following equation:

$$\begin{array}{l} s(i)\;=\;\frac{b\left(i\right)-\;a\left(i\right)}{\max\left\{a(i),b(i)\right\}} \end{array}$$

where *a(i)* is the average dissimilarity of element *i* with the other elements within the same cluster and *b(i)* is the lowest average dissimilarity of element *i* between the other clusters which do not contain *i*. The silhouette index ranges from −1 (bad element to cluster agreement) to +1 (good element to cluster agreement). The average silhouette values were computed for all the vertices and setting the number of *k* centroids from 2 to 7. The two local maxima (3 and 6) for dataset 1 were chosen as number of clusters for the *k*-means algorithm. Qualitatively inspecting the results showed that three clusters corresponded to two cortical layer clusters (in gray matter) and one noise cluster (almost invariably located at tissue boundaries) and similarly six clusters corresponded to four cortical layer clusters in gray matter and two noise clusters. Therefore, we refer to these results as the two-layer cluster and four-layer cluster results, respectively. Since the centroid locations are always initialized at random positions, the clustering algorithm was run 100 times and the best solution (i.e., the one which minimized the sum of differences between the feature space and the centroids) was kept. No spatial proximity information was used in the feature matrix for the layer-clustering procedure, i.e., it was based on absolute (in the total cortical diffusivity) and orientation dependent diffusion information (in the other features) only.

For visualization purposes, the layer cluster maps were interpolated using mode filtering. First, the original layer cluster map was interpolated to three times the resolution using a nearest neighbor interpolation method as implemented in Matlab (R2010b, The MathWorks, Natick, Massachusetts, USA). Then, in the interpolated volume, every element was substituted with the mode of the values contained in a 5 × 5 × 5 window.

To assess reproducibility between the two datasets and correspondence between histology and dMRI-based layer clustering, cross-table contingency analysis and Chi-Squared statistics were used to evaluate its significance, as implemented in Matlab (R2010b, The MathWorks, Natick, Massachusetts, USA). After myelo- and cytoarchitectural layers were delineated, the corresponding layer number was assigned to every pixel of the histological section. Then, the resolution of the aligned histological sections was downsampled to match that of the dMRI datasets (340 μm isotropic), using a nearest-neighbor method as implemented in Matlab (R2010b, The MathWorks, Natick, Massachusetts, USA), giving an appropriate number of degrees of freedom for Chi-Square statistics when performing the cross-table contingency analysis.

### Cortical area demarcation

The implementation of the area boundary demarcation technique uses diffusivity indices defined across cortical depth and a sampling grid defined in the 3 dimensional space which comprised the tissue sample. The approach is based on Schleicher et al. (2000) and uses a local stepping procedure based on Mahalanobis distances, extended to a 3D cortical sampling grid. To implement the area demarcation technique, a new grid was defined in gray matter. The three dimensional grid samples the cortical volume both radially at 9 equi-spaced depths and tangentially in the latero-medial and antero-posterior direction in 120 μm steps. This approach is capable of reliably following folded cortex at different relative proportional depths (of 0–100%, see above). Furthermore, the streamlines which connect the two outer cortical boundaries connect corresponding points within the depth sampling grids across multiple cortex depth planes, forming what we refer to as cortical depth profiles. The 100 × 200 × 9 grid was positioned to cover the crown of the precentral gyrus and the precentral sulcus (Supplementary Figure 3). The grid was formed by laying down a 200 discrete point streamline over the surface reconstruction in the anterio-posterior direction. This streamline was then replicated in the latero-medial direction 99 times, at 170 μm spacing and in the cortical depth direction over the 9 surfaces. To increase the sensitivity of the boundary algorithm, the ADC-based indices were averaged across 10 latero-medial grid positions, resulting in a 10 × 200 × 9 three-dimensional grid. The areal classification was performed independently of the layer classification on a single diffusion information vector per cortical surface location, which contains all diffusion feature information along cortical depth. The feature space consisted of the first four (i.e., 1st, 2nd, 3rd, and 4th order) moments around the mean of each of the ADC indices (total, radial, and tangential) calculated along the cortical depth profile, using the sampled grid. Therefore, 12 different coefficients were defined at every grid point, forming a local coefficient profile. Stepping along the antero-posterior direction, the Mahalanobis distance between profiles in two blocks of N contiguous profiles was computed:

$$\begin{array}{l} D=\left(\bar{X_{1}}-\bar{X_{2}}\right)C^{-1}{\left(\bar{X_{1}}-\bar{X_{2}}\right)}^{\prime } \end{array}$$

where ${\bar{X}}_{1}$ and ${\bar{X}}_{2}$ are the mean feature vectors of every block at each integration step and *C* is the pooled covariance matrix (Amunts et al., 2010). The block size N was varied between 12 and 24 profiles in each block to ensure stability of the pooled covariance matrix and reproducibility of the identified boundary over spatial scales. Significant boundaries were identified using Hotelling's *T*^2^ statistics corrected for multiple comparisons using Bonferroni correction. Finally, the total number of significant counts was computed, summing the number of significant candidate boundaries at each profile block size (spatial scale; see **Figure 4**, lower left inset), across the 10 different averaged lattices. These total numbers of counts were thresholded at 4.5 standard deviations (red lines in **Figure 4**) to demarcate area boundaries in both datasets.

## Results

### Automatic demarcation of cortical layers

**Figure 1:**
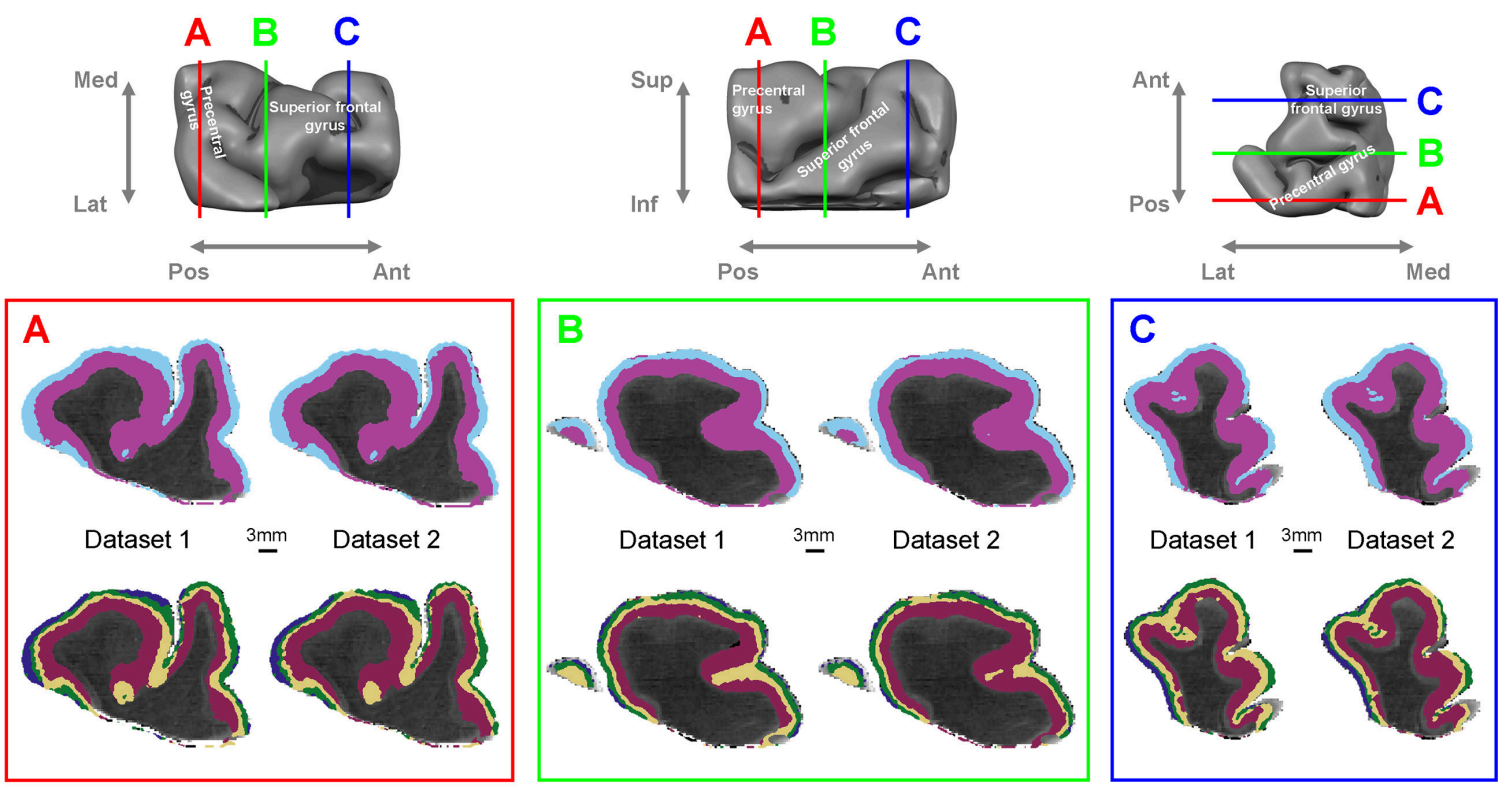
**Automatic layer classification from high-resolution dMRI for dataset 1 and 2.** (Top row): macro-anatomical description of the tissue sample and virtual section plane locations. (Bottom row) (A,B,C): coronal sections (viewed from the anterior side) through the 3D dMRI data showing automated cortical layer classification results overlaid on mean diffusivity (MD) maps. The three panels show the two-layer cluster (top row) and the four-layer cluster results (bottom row).

Figure 1 shows the results of the automated layer classification algorithm applied to the full extent of cortex in the tissue sample, containing motor and premotor areas in precentral and superior frontal gyri. The classification results for the two different datasets (dataset 1 and dataset 2) show a very high degree of correspondence, indicating strong reproducibility. Silhouette analysis for the optimal number of clusters in the gray matter gave two local optima, the first for two layer clusters and the second for four layer clusters (Supplementary Figure 4). The upper row in panels A, B, and C shows the two layer cluster (two-cluster) result, whereas the lower row shows the four layer cluster (four-cluster) result. It can be seen that the two-cluster result clearly identifies a boundary between superficial and deep cortical layers in a reproducible way between dataset 1 and 2. In the selected slices, the classification algorithm marks two clusters maintaining a consistent boundary and their relative depth ordering over most of the studied expanse of cortex. This is an important finding, given that no information on spatial cortical location (such as cortical depth or tangential proximity to other voxels) was given to the algorithm; it operated *only* on orientation dependent diffusion characteristics in the local cortical coordinate frame. Note that the light blue superficial band disappears in some locations which may indicate inaccurate classification, but this may also result from the shallow or highly oblique slicing of gyral/sulcal walls in the depicted section plane. The four-cluster result in the lower row shows that the information in the diffusion data supports even further subdivision into four different clusters in many parts of the cortical ribbon. To quantitatively investigate the reproducibility between datasets, we performed cross-table contingency analysis and Chi-Squared statistical analysis. Cross-table contingency analysis between the two- and the four-cluster results for the same datasets showed that the four-cluster result subdivides each of the two classes from the two-cluster results, retaining the two-cluster boundary. Again, these clusters maintain their relative depth ordering almost everywhere. While only a subset of the clusters is present at certain locations, most of the gray matter that is nearly perpendicularly cut by the displayed section planes is subdivided into four layer clusters with only the outermost and thinnest (dark blue) layer cluster sometimes missing identification. Automatically labeled clusters broaden and narrow depending on their location particularly in gyral crowns or sulcal fundi, reflecting known anatomy (Bok, 1929).

**Figure 2:**
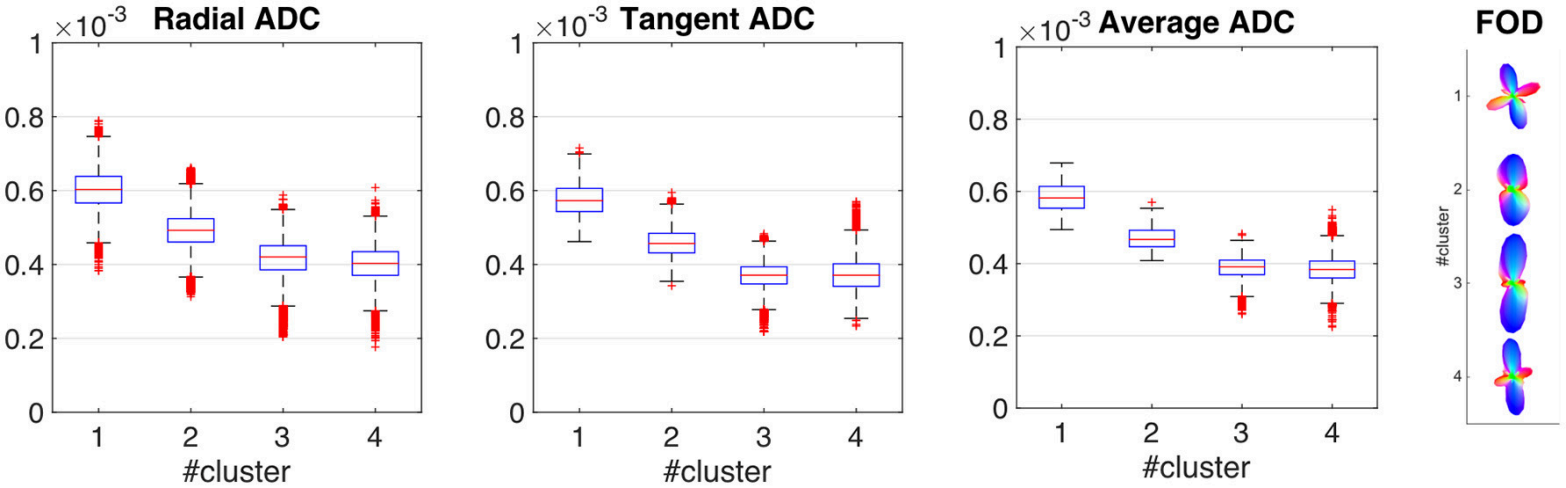
**Cluster profiles.** Distribution of each ADC related feature within each cluster, summarized using boxplots (first three panels). The other 28 features (i.e., the spherical harmonics coefficients of the ADC profile obtained using a maximum harmonic order of 6) are represented using the fiber orientation distribution obtained when deconvolving the average ADC profile for each cluster with a single fiber response function (rightmost panel). The response function was estimated from the 300 voxels with the highest FA within white matter.

Figure 2 shows the distribution of each ADC related feature within each cluster, summarized using boxplots. The decrease in ADC when moving from the pial surface to the WM/GM boundary is consistent with previous studies (e.g., Kleinnijenhuis et al., 2013). The other 28 features (i.e., the spherical harmonics coefficients of the ADC profile obtained using a maximum harmonic order of 6) are represented using FOD obtained by deconvolving the average ADC profile for each cluster with a single fiber response function (Tournier et al., 2004, 2007). Clusters 1 and 4 (most supragranular and infragranular clusters) show clear tangential component, while all clusters present a clear radial component. A small tangential component is still present in cluster no. 3 while cluster 2 shows an increased dispersion which might be associated with increased fiber dispersion possibly due to a lower myelination in more superficial layers.

**Figure 3:**
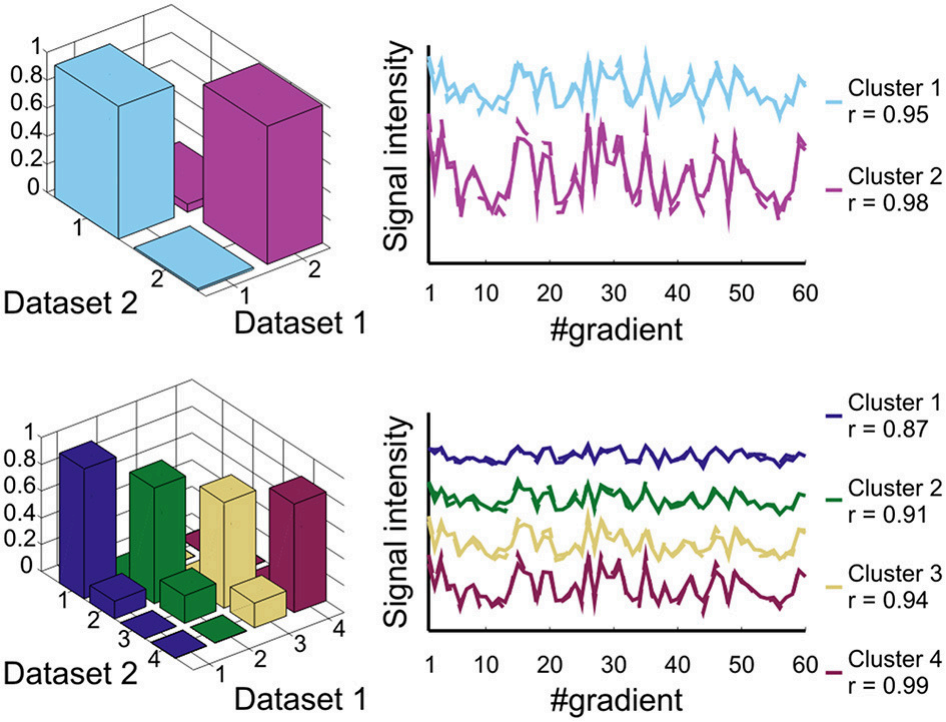
**Reproducibility analysis.** Two-layer (top row) and four-layer result (bottom row). Left column: cross-table analysis of dataset 1 against dataset 2; right column: correlation analysis of the layer cluster signal profile between dataset 1 (solid line) and dataset 2 (dashed line).

The reproducibility of the layer classification result between the two separate datasets is shown in Figure 3. The correspondence (in terms of the cross-table fraction) of the spatial clustering results between dataset 1 and dataset 2 is larger than 0.94 (mean = 0.97) for the two layer cluster analysis and larger than 0.79 (mean = 0.86) for the four layer cluster analysis. The Chi-Squared contingency analysis showed a highly significant association between the two datasets for both the two layer cluster result (χ^2^ = 5.18e + 005, *p* < 0.001) and the four layer cluster result (χ^2^ = 7.78e + 005, *p* < 0.001). The degree of similarity of the signal profile (rotated to the cortical frame) between the two datasets for corresponding clusters is very high with correlations of 0.95 and 0.98 for the two layer cluster analysis result and between 0.87 and 0.99 for the four layer cluster analysis. This shows that the distinctiveness of the signal profile, which leads to the identification of the different layer clusters, is also highly reproducible.

### Histological validation of automated layer clustering

**Figure 4:**
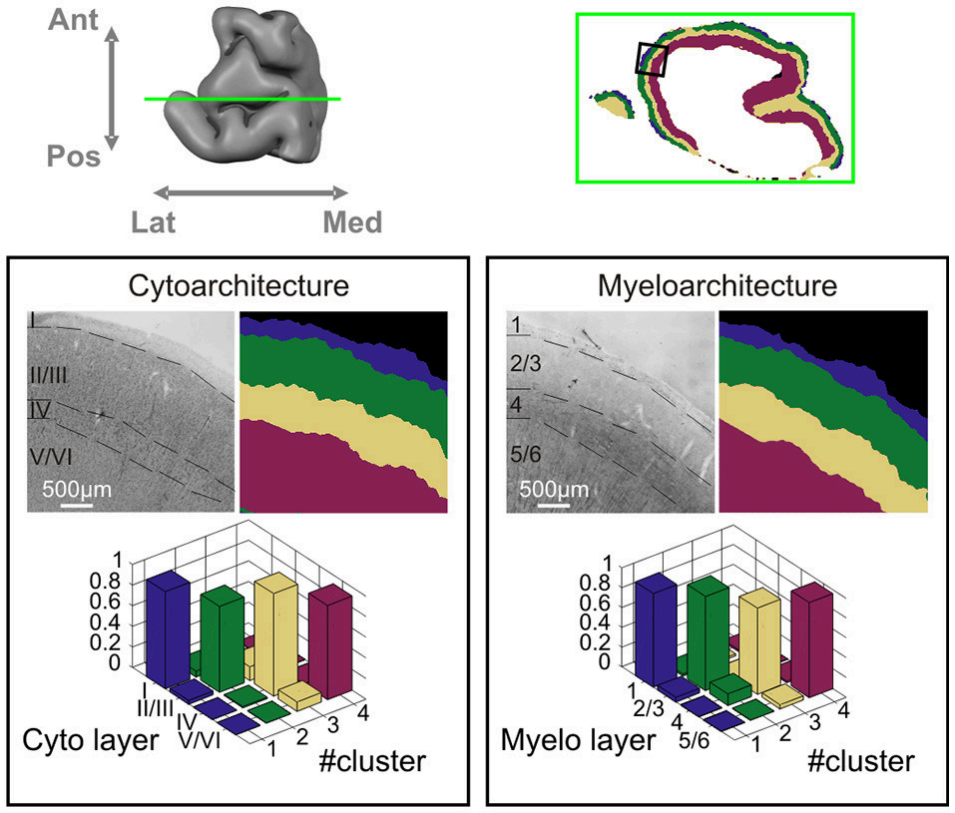
**Histological validation.** Correspondence between the four-layer cluster result of dataset 1 and histology on the same tissue block. Upper row: location of the coronal section. Lower panels: correspondence to cytoarchitecture (left) and myeloarchitecture (right). For each panel, the upper row depicts histological classification of layers and dMRI layer cluster result, and bottom row shows the cross-table contingency analysis between histology and dMRI based layer clustering.

We validated the automatic classifications by histological sectioning and staining of the same tissue sample, as shown in Figure 4. The laminar organization obtained from the layer classification is shown along with cytoarchitectural and myeloarchitectural classifications of the same tissue. Quantitative correspondence between automated clustering and histology is again reported by cross-table contingency analysis. A very high correspondence is found between both cytoarchitecture and myeloarchitecture and automatic clustering based on dMRI. For myeloarchitecture classification when grouping together both layers 2 and 3 and layers 5 and 6 into two-layer complexes the correspondence is larger than 0.87 (mean = 0.93, χ^2^ = 127.02, *p* < 0.001). For cytoarchitecture, again when grouping together layers 2 and 3 and layers 5 and 6, the correspondence is larger than 0.83 (mean = 0.92, χ^2^ = 110.22, *p* < 0.001). This shows there is a strong match between the layer boundaries identified by the algorithm and both the myeloarchitectural and the cytoarchitectural ones identified in the histology sections. As we expect dMRI signal to be more sensitive to oriented neurites, we use roman numerals, as is customary in myeloarchitectural notation, to label cortical layers in the following. A detailed analysis of the architectural correspondence reveals layer cluster 1 to correspond to cortical layer 1, cluster 2 to layers 2/3, cluster 3 to layer 4 (when present, i.e., in premotor cortex) and, finally, cluster 4 to layers 5/6. That is, layers 1 and 4 were mostly identified as an individual cluster, whereas cortical layers 2 and 3 and cortical layers 5 and 6 are each clustered together.

### Automatic boundary detection of cortical areas

**Figure 5:**
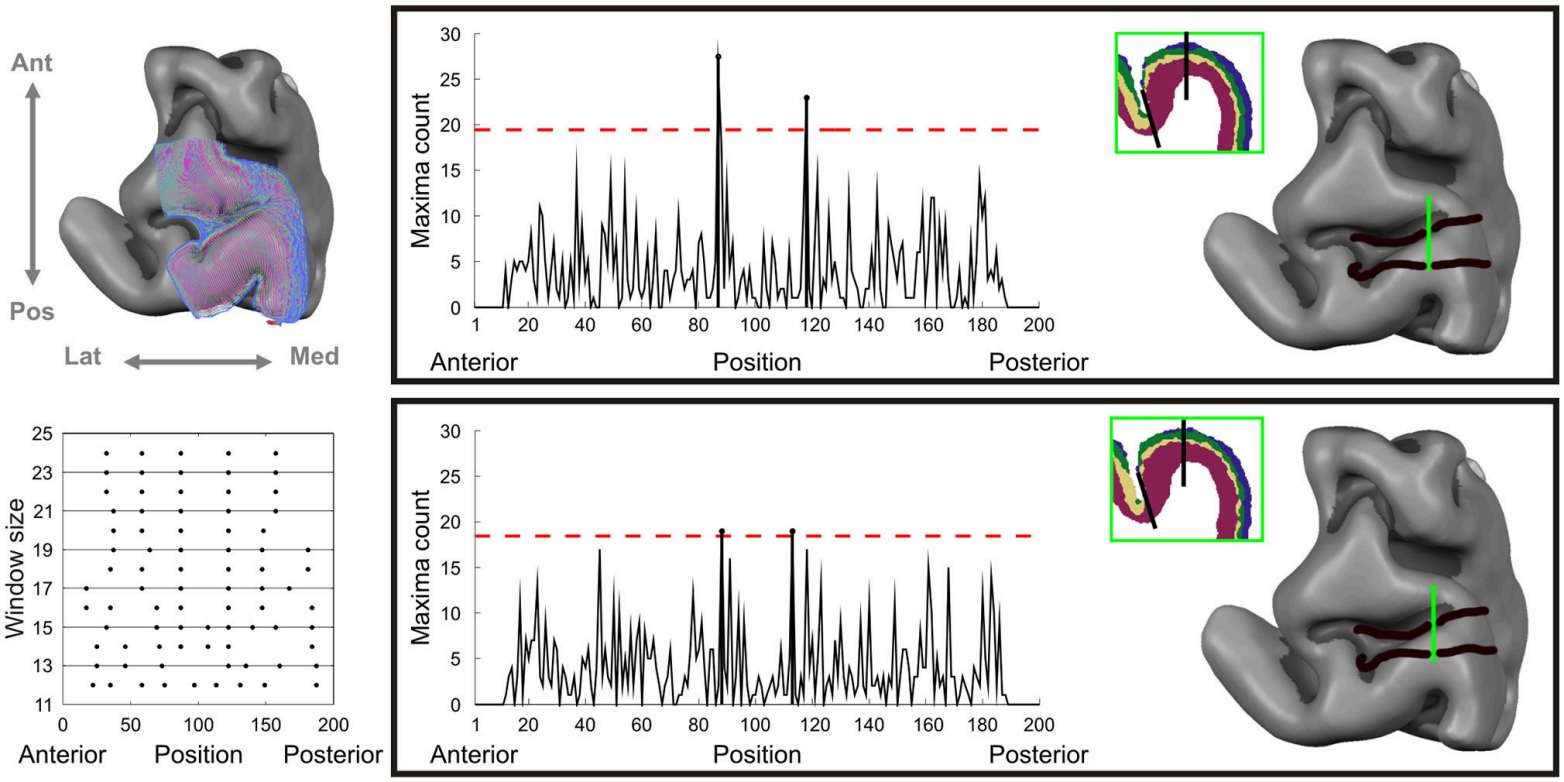
**Automated observer independent cortical area boundary detection on dMRI data.** Left column, upper inset: cortical depth sampling grid straddling the precentral gyrus and sulcus. Left column, lower inset: thresholded Hotelling's statistics for significant detected boundary over different window sizes and cortical anterio-posterior position. Right panels for dataset 1 (top) and dataset 2 (bottom): on the left the significant boundary index summed over scales with the horizontal line corresponding to 4.5 standard deviations and on the right the identified super threshold cortical area boundaries on the sampling grid.

Having examined cortical lamination patterns, we investigated whether we were also able to architecturally demarcate different cortical areas from dMRI data in gray matter. Figure 5 shows that the signature of dMRI signals over cortical depth can be used to detect boundaries on the cortical surface with abrupt changes in depth dependent diffusion characteristics. The dMRI-based demarcation is based on local Mahalanobis distances between feature vectors defined over cortical depth. In this approach we move along a three-dimensional intra-cortical grid (with one depth dimension and two tangential dimensions) from posterior to anterior along the precentral gyrus and sulcus (Figure 5, upper left), collecting information on local feature vector distances. The points of the largest local feature vector distances over multiple window sizes (spatial scales) identify cortical area boundaries (Figure 5, lower left). Two boundaries are consistently identified in both datasets. The first is close to the crown of the precentral gyrus, the second and most prominent one is located in the fundus of the precentral sulcus (Figure 5, right panels). When observing the automatic classification of cortical layers at these boundaries, local changes in layering can be seen as the underlying cause for boundary detection (insets in Figure 5). At the boundary located more posteriorly, on the crown of the precentral gyrus, we observe a gradual widening of layer cluster 4, corresponding to the deep layers 5 and 6. At the boundary located more anteriorly in the precentral sulcus, it is rather cluster 3, corresponding to layer 4, which broadens somewhat abruptly.

## Discussion

There has been a century-old dominance of the cytoarchitectonic classification of human cortex, mostly based on Brodmann's cortical parcellation scheme (Brodmann, 1909). This scheme is mainly based on the analysis of shape and organization of neuronal cells as observed using Nissl stained histological sections. Using this technique, Brodmann was capable of mapping a total of 52 areas on the cortices of several mammals, including a human sample. So far, Brodmann's classification of cortical areas has been the most widely adopted one when reporting the results of several neuroimaging studies (Zilles and Amunts, 2010). Other parcellation schemes are available, such as those based on cortical myeloarchitecture (Vogt, 1910; Nieuwenhuys, 2013). These schemes look at the myelinated fibers densities and orientations to define boundaries between adjacent cortical areas. Recently, new classifications are emerging, for instance based on neurotransmitter receptor architecture (Amunts et al., 2010). Moreover, new ways to probe the cyto- and myeloarchitectural of the brain have recently been developed, such as polarized light imaging (Axer and Keyserlingk, 2000; Axer et al., 2001; Axer H. et al., 2011; Axer M. et al., 2011) and optical coherence tomography (Wang et al., 2011; Magnain et al., 2015). These techniques allow to probe cyto- and myeloarchitecture in three dimensions at the resolution of few microns.

Here, we investigated the demarcation of cortical lamination patterns over large expanses of human motor cortex by applying a clustering algorithm to very high-resolution dMRI signal characteristics. The diffusion characteristics were derived from high isotropic *spatial* resolution (340 μm) and high *angular* resolution dMRI data which is rotated into the local cortical coordinate frame comprising radial and tangential orientations to the cortical surface.

The two layer cluster result demarcates a clear boundary between superficial and deep cortical layers stable over multiple cortical areas and measurements under different diffusion conditions. Considering the histological validation results (Figure 4) there is a very robust identification of what Vogt (1910) already called the inner and outer main cortical zones (the “Innere” and “Äußere Hauptzone”), with the superficial zone containing layers 1, 2, and 3 and the deep zone containing layers 4, 5, and 6. Beyond that, the four-cluster result subdivides each zone further into layer clusters, corresponding to layer 1, a layer 2/3 complex, layer 4, and a layer 5/6 complex. Although less consistent than the two-cluster result, the more detailed four-cluster result shows a remarkable consistency over long stretches of cortex and a high reproducibility with cross table correspondence fractions in the 0.8–0.9 range.

As shown in Figure 1, there were interruptions of some layers (particularly layer 1) along the length of the cortex in the four layer cluster result. The most likely cause of this is partial volume effects since our high spatial resolution of 340 μm is still only just sufficient to distinguish the very thin layer 1, comprising only about 100–200 μm, in certain portions of the sampled tissue. We are still able to identify this structure of about half a voxel width because the neighboring embedding fluid has no orientation dependence (i.e., isotropic diffusion). Thus, adding its signal decreases orientation dependent contrast-to-noise of the tissue contribution but does not destroy the important orientation dependent signal structure in the voxel. Even further increases of spatial resolution can alleviate this issue, and could potentially also help uniquely identify thin deeper layers. An interesting future challenge is achieving such higher resolution while maintaining, or even extending, spatial tissue coverage and angular diffusion resolution (i.e., number of diffusion encoding directions).

Our histological analysis revealed that the automatic clustering identified layer 1, layers 2/3, layer 4 (when present, i.e., in premotor cortex), and layers 5/6 consistently in the same clusters. In correspondence with the generally accepted six-layer structure of neocortex, we found an equally high level of agreement of the automatic dMRI clustering with the cytoarchitectural and myeloarchitectural classification of the cortical layers. Agreement of the dMRI clustering with myeloarchitecture is qualitatively slightly higher (i.e., marginally higher cross-table fractions) but correspondence for both is very high and statistically significant. Historically, Vogt argued that it is only the sub-classifications of the six layers that can be different and in particularly more detailed in myelo- than in cytoarchitecture. In addition, Hellwig (1993) found that when limited to the six canonical neocortical layers, myeloarchitecture can be predicted from cytoarchitecture, confirming their basic agreement. Because of the basic orientational contrast of dMRI, it is likely that the automatic layer classification presented here is mostly sensitive to myeloarchitecture. For instance, layer 1 and 4 are likely identified because of the strong orientational contrast provided by their characteristic tangential plexus of fibers. A comment should be made regarding cyto- and myeloarchitectural layer 4. This layer was identifiable in most of the histological sections as the tissue sample mainly comprises the precentral and superior frontal gyri. These areas classically belong to premotor cortex, where a thin granular layer can be identified in cytoarchitectural stains, in contradistinction to primary motor cortex which lacks layer 4 though this anatomical feature has recently been debated (though this anatomical feature has recently been debated, see Geyer et al., 2000; García-Cabezas and Barbas, 2014; Barbas and García-Cabezas, 2015). The presence of the cluster preferentially associated to layer 4 in the paracentral lobule region as well as in the dorsal part of the precentral gyrus might reflect the fact that, despite the apparent lack of a clear cytoarchitectural granular layer, the dMRI signal captures the preferential tangential orientation of myelinated fibers between supra- and infragranular layers (Vogt, 1919).

The area classification results suggest that the orientation sensitivity of dMRI potentially contains enough information to automatically classify architecturally distinct cortical areas. Two boundaries were identified, the first marking a boundary close to the crown of the precentral gyrus, the second a boundary in the fundus of the precentral sulcus. These are similar to the established motor cortex parcellation originally described by Vogt (1910) and later confirmed by Sanides (1962) who divided the precentral and superior frontal gyrus into areas 42, 39, and 38. Indeed, the boundary between areas 42 and 39 lies close to the crown of the precentral gyrus in the superior part of the brain, which is also demarcated by our posteriormost boundary. The boundary between areas 38 and 39 is located close to the fundus of the precentral sulcus, corresponding very well to our anteriormost boundary. Here broadly speaking, area 42 agrees with Brodmann's area 4 and primary motor cortex while 38 and 39 are a further subdivision of Brodmann's area 6 and a part of premotor cortex. Furthermore, the identified boundaries agree well with observer-independent cytoarchitecture (Geyer et al., 1996; Fischl et al., 2008) which was recently also confirmed using *in-vivo* myelin mapping (Glasser and Van Essen, 2011). However, it should be noted that this contrasts with findings that, although Brodmann's area 4 correlates well with Sanides' motor area 42, it tends to cover only small portions of the exposed surface of the precentral gyrus (Rademacher et al., 2001) and mostly in the dorsal aspects (investigated here). That combined cyto- and myelo-architectonic study also highlights the intersubject variability of area boundaries and the poor correspondence to macroanatomical gyral or sulcal landmarks.

A limitation of this proof-of-principle methodological study is that it uses only one post mortem tissue sample. Despite having shown a high degree of reproducibility and robustness of the results when scanning the same tissue twice under different conditions, application, and generalization of the results over the wider human cortex will require future tests of the proposed approach on other samples of different cortical regions. Moreover, the resolution that could be achieved in this study is not optimal to disentangle single cortical layers. Hardware improvements and new MR sequences might improve this limitation and effectively allow dMRI-based segmentation of single layers, although it is crucial to achieve very high resolution over large fields-of-view that encompass large stretches of cortex. This is very important as dMRI can provide specific information about the underlying tissue microstructure such as radial and tangential fiber organization. Another limitation of the study is that the proposed methods to automatically demarcate cortical layers and areas using dMRI derived indices both rely on a cortical sampling algorithm based on the Laplace method, which has recently been shown to have a curvature-related bias in sampling layers over the depth of the cortex. A more anatomically accurate method has been developed to sample the cortex based on an equi-volume cortical sampling strategy (Waehnert et al., 2014). Both the layer classification approach and the area boundary identification approach proposed here could be adapted to alternative cortical depth sampling strategies. It should be noted, however, that the proposed algorithms are relatively insensitive to curvature-related bias. This is because the main bias is in the relative depth location of layer, rather than in the local coordinate system. Furthermore, the area boundary identification looks only at local changes in features over depth, such that it compares only feature vectors with a very similar depth bias. Another limiting factor that might influence the results of the current work lies in the choice of features. These are mostly based on the ADC profile and on directional information as derived from the spherical harmonics coefficients. Moreover, the present study is that the data was acquired using only a single b-value (3000 s/mm^2^). Recent work (Jbabdi et al., 2012) has shown the benefit of using multi-shell data when modeling the dMRI signal. Combining high SNR—low angular contrast (low b) with low SNR—high angular contrast (high b) shells allows to reduce overfitting problems when modeling the dMRI signal. Further work needs to be done to extend the proposed approaches making use of different microstructural indices (e.g., Assaf and Basser, 2005; Zhang et al., 2012) that can be derived from dMRI and that might help in segmenting cortical layers and areas better.

## Author contributions

MB, AO, DB, KM, NS, RGa, RGo and AR designed research; MB, AO, AS, RGa, and AR acquired the data; DB, KM, AC, JF, HB, NS, RGa, RGo and AR provided hardware/software support for data acquisition and analysis; MB and AR analyzed the data; MB and AR wrote the paper.

### Conflict of interest statement

The authors declare that the research was conducted in the absence of any commercial or financial relationships that could be construed as a potential conflict of interest.
