# Supplementary material for: Automatic Segmentation of Human Cortical Layer-Complexes and Architectural Areas Using Ex vivo Diffusion MRI and Its Validation
Source: Front Neurosci. 2016 Nov 10;10:487. doi: 10.3389/fnins.2016.00487 (PMC5102896; doi:10.3389/fnins.2016.00487)
Supplement: Supplementary file 1 [file DataSheet1.docx]

Supplementary Material

Automatic segmentation of human cortical layer-complexes and architectural areas using ex vivo diffusion MRI and its validation

Matteo Bastiani, Ana-Maria Oros-Peusquens, Arne Seehaus, Daniel Brenner, Klaus Möllenhoff, Avdo Celik, Jörg Felder, Hansjürgen Bratzke, Nadim Jon Shah, Ralf Galuske, Rainer Goebel and Alard Roebroeck

*** Correspondence:** A. Roebroeck: [a.roebroeck@maastrichtuniversity.nl](mailto:a.roebroeck@maastrichtuniversity.nl)

# Supplementary Figures

#
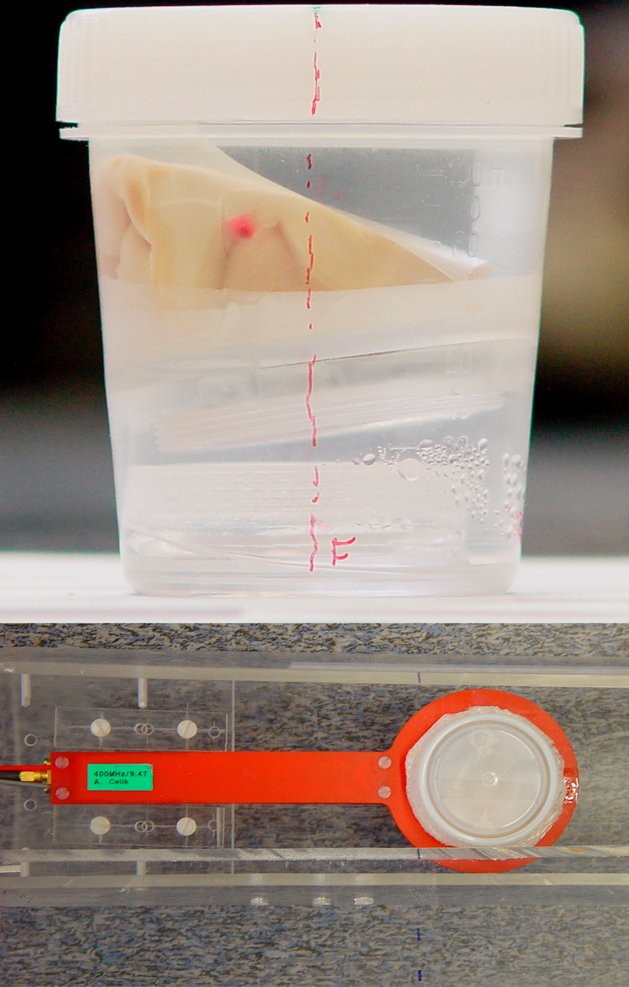


**Supplementary Figure 1.** MR acquisition setup. Top: tissue block in its plastic container immersed in the fixation solution; a line was drawn on the container for subsequent reference for histological dissection. Bottom: container inserted in the 7cm loop coil used for RF transmission and signal reception.


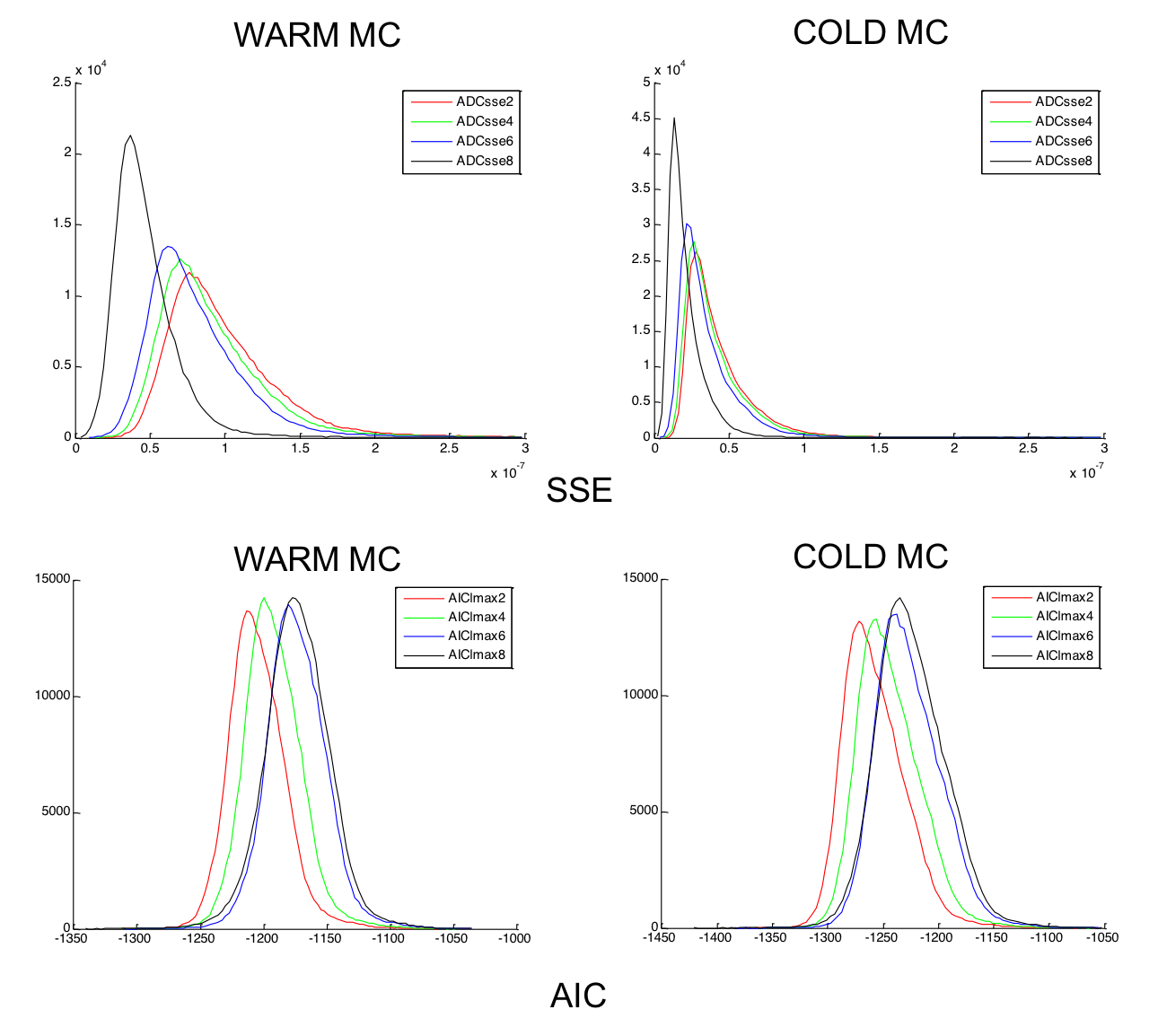


**Supplementary Figure 2.** Histograms of sum of squared errors (SSE) and Akaike information criterion (AIC) for all voxels in gray matter and for both data sets (‘WARM MC’ and ‘COLD MC’) using an ordinary least square approach to estimate the spherical harmonics coefficients. Note that the SSEs and AICs cannot be compared between datasets, because they have been computed in the arbitrary units of the respective raw datasets. Within dataset comparisons can be made.


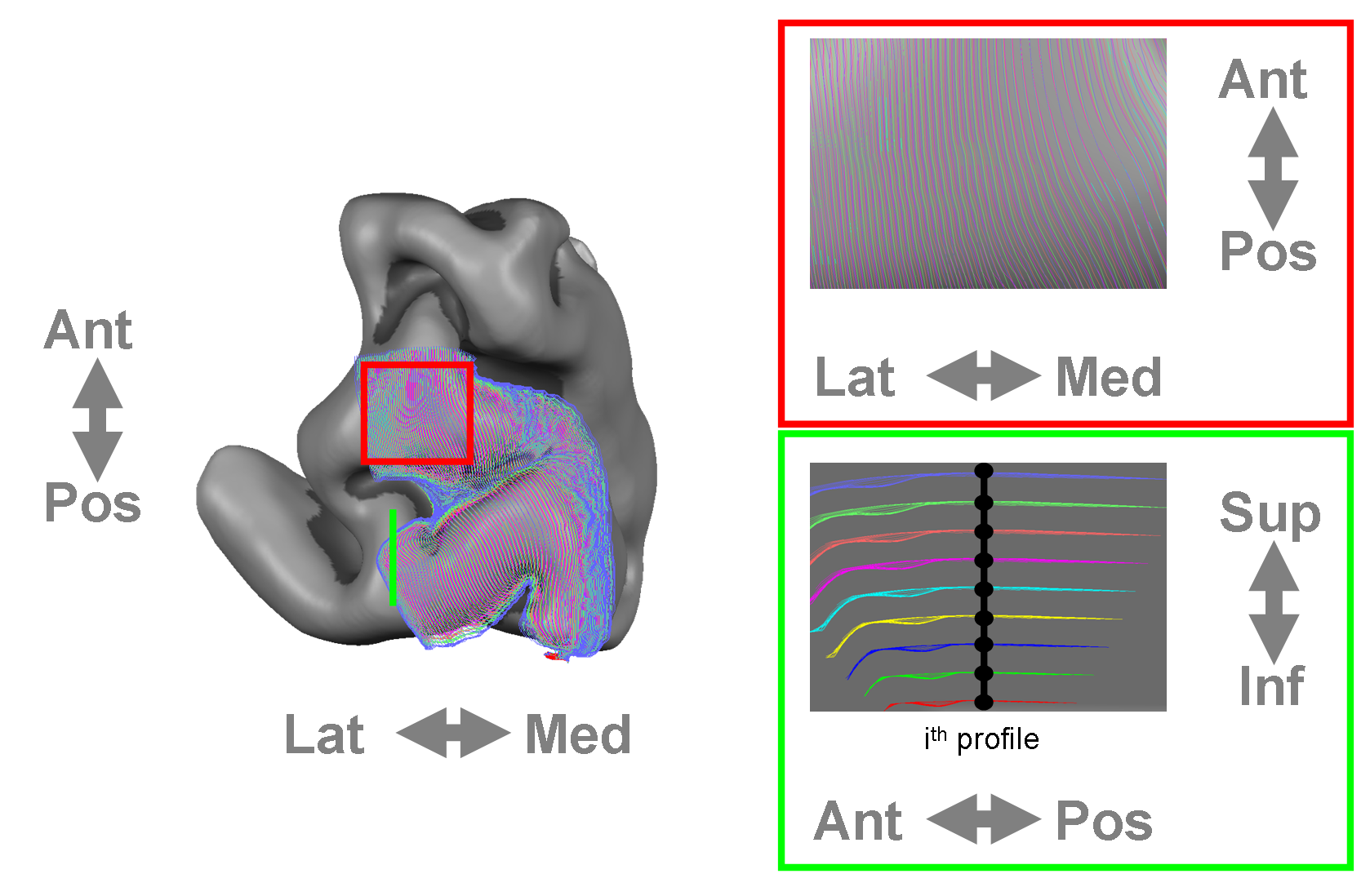


**Supplementary Figure 3.** Three dimensional grid for area boundaries demarcation. The grid is superimposed to the sampled tissue (left). Red inset: streamlines of 200 equi-spaced steps sampling along the antero-posterior direction. Green inset: sagittal view of the 9 cortical meshes sampling the gray matter volume. The i^th^ profile is defined radially along the cortical normal at every intersection point.

**
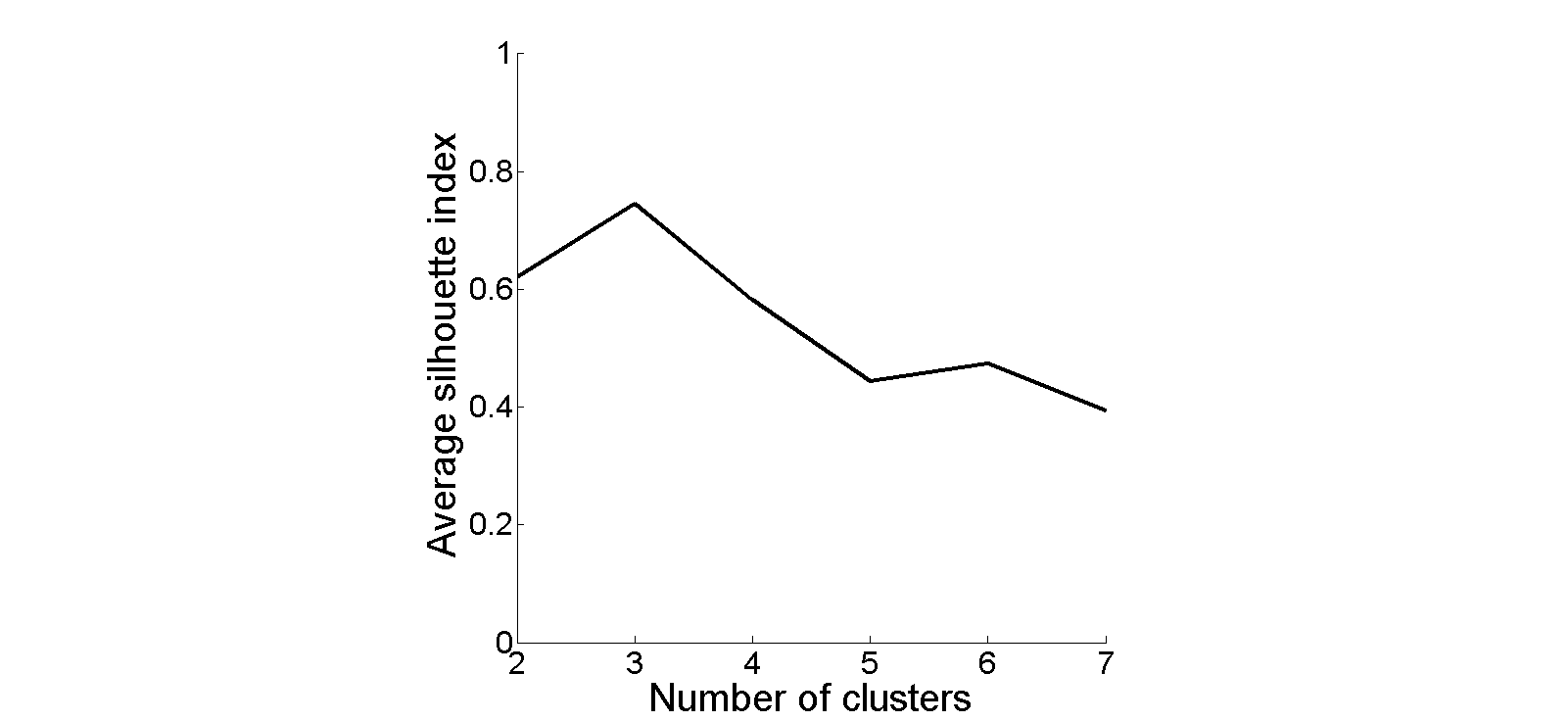
**

**Supplementary Figure 4**. Silhouette analysis for the optimal number of clusters. In dataset 1 the silhouette analysis shows a maximum at 3 clusters (corresponding to 2 clear layer clusters and 1 noise cluster) and another (local) maximum at 6 clusters (corresponding to 4 clear layer clusters and 2 noise clusters)
